# Associated factors and outcomes of crossover from a laser sheath to a bidirectional rotational mechanical sheath during transvenous lead extraction

**DOI:** 10.1002/joa3.12929

**Published:** 2023-09-26

**Authors:** Tsuyoshi Isawa, Taku Honda, Kazuhiro Yamaya, Shigeru Toyoda, Masataka Taguri

**Affiliations:** ^1^ Department of Cardiology Sendai Kousei Hospital Sendai Japan; ^2^ Department of Cardiovascular Surgery Sendai Kousei Hospital Sendai Japan; ^3^ Department of Cardiovascular Medicine Dokkyo Medical University Mibu Japan; ^4^ Department of Health Data Science Tokyo Medical University Tokyo Japan

**Keywords:** device crossover, Evolution RL sheath, Evolution Shortie RL sheath, GlideLight laser sheath, lead extraction

## Abstract

**Background:**

During transvenous lead extraction (TLE), a GlideLight laser sheath (Philips) cannot always be advanced over the lead, and crossover to the Evolution system (i.e., an Evolution RL sheath or Evolution Shortie RL sheath [Cook Medical]) is required. We aimed to determine the associated factors and outcomes of such device crossover.

**Methods:**

This observational study included 112 patients who underwent TLE. The patients were divided into crossover and non‐crossover groups. Outcomes and associated factors of crossover were evaluated.

**Results:**

Overall, 57 (50.9%) patients required crossover to the Evolution system (crossover group), whereas 55 (49.1%) patients did not require crossover (non‐crossover group). Clinical success rate was similar between the two groups (98.3% vs. 100%; *p* = 1.00). No major intraprocedural complications related to powered sheaths occurred. Multivariate logistic regression analysis results showed that dwell time of the oldest extracted lead (per year) (odds ratio [OR]: 1.18, 95% confidence interval [CI]: 1.02–1.36; *p* = .026), number of leads extracted per procedure (OR: 7.23, 95% CI: 1.74–29.99; *p* = .007), and use of a femoral approach (OR: 21.09, 95% CI: 2.33–190.67; *p* = .007) were associated factors of crossover. The cutoff for crossover was 7.7 years from the implant (sensitivity 90.5%, specificity 64.9%, area under the curve 0.80).

**Conclusions:**

Both groups showed a high rate of clinical success. Switching to the Evolution system may facilitate a safe and effective TLE when a laser sheath does not advance despite laser activation.

## Graphical abstract

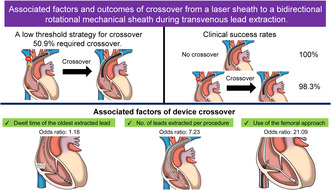

## 1 INTRODUCTION

The increased use of cardiac implantable electronic devices over the past decade has led to increased transvenous lead extraction (TLE) worldwide.^1^ In addition to nonpowered sheaths, such as polypropylene sheaths (Byrd Polypropylene Dilator, Cook Medical),^2^, ^3^, ^4^ powered sheaths are used for TLE. The commonly used powered sheaths are the GlideLight laser sheath (Philips) and the bidirectional rotational Evolution RL sheath/Evolution Shortie RL sheath (Cook Medical). Laser sheaths are more commonly used^5^ due to good clinical outcomes^6^ and operators' familiarity with laser sheaths resulting from the earlier introduction into the market compared with that of an Evolution RL sheath or an Evolution Shortie RL sheath (i.e., Evolution system). Although the first‐line use of laser sheaths is more common, these sheaths cannot always be advanced over the lead, and crossover to the Evolution system is required. However, few studies have investigated the associated factors and outcomes of crossovers. Therefore, we conducted a retrospective observational study to determine the associated factors and outcomes of crossover from a GlideLight laser sheath to the Evolution system during TLE.

## 2 MATERIALS AND METHODS

### 2.1 Patient selection

Consecutive patients who required TLE due to infection, lead malfunction, or device upgrade at the Sendai Kousei Hospital from July 2019 to June 2023 were screened. The exclusion criteria were as follows: Leads removed (i) by manual traction alone (no powered sheaths) and (ii) by first‐line use of the Evolution system. The patients who required crossover were compared with those who did not.

### 2.2 Heart team evaluation

Every patient was evaluated by a heart team comprising dedicated cardiologists, cardiac surgeons, anesthesiologists, and cardiac perfusionists. Indications for TLE were based on the 2009 and 2017 Heart Rhythm Society consensus documents^7^, ^8^ and the Japanese Circulation Society/Japanese Heart Rhythm Society 2019 guidelines.^9^

### 2.3 Lead extraction procedure

The preprocedural evaluation included routine laboratory studies, electrocardiography, chest X‐ray, spirometry, transthoracic echocardiography, and bilateral subclavian venography. Blood and wound cultures were collected from all infected patients. All procedures were performed in a hybrid operating room with the patient under general anesthesia with transvenous temporary pacing, invasive arterial blood pressure monitoring, transesophageal echocardiography, and immediate onsite cardiovascular surgical coverage. TLE was conducted by the heart team and primarily performed using a superior approach. After the pocket was opened and drained and the generator was removed, the lead adhesion close to the clavicle was dissected to secure the traction in line with the subclavian vein. If present, the active fixation mechanism was retracted, and manual traction was attempted. When manual traction was ineffective, the superior approach using a GlideLight laser sheath coupled with a locking stylet (LLD lead locking device; Philips) was employed. A 12‐Fr laser sheath was initially used for a pacemaker lead and a 14‐Fr one was initially used for a lead of an implantable cardioverter defibrillator, respectively. The outer sheath for the laser was not used. If this approach did not work, the Evolution RL sheath or the Evolution Shortie RL sheath (to the venous entry site) was used. In case of failed advancement with the 12‐Fr laser sheath, it was switched with a 9‐Fr Evolution RL or a 9‐Fr Evolution Shortie RL sheath. In case of failed advancement with the 14‐Fr laser sheath, it was switched with an 11‐Fr Evolution RL or an 11‐Fr Evolution Shortie RL sheath. Manual compression on the venous entry site was always performed by an assistant to reduce bleeding during the switch from a laser sheath to an Evolution system. Notably, the Evolution system was introduced when a laser sheath could not be advanced over the lead after an activation time of 5–6 s. In cases wherein multiple lead extractions were required and advancing the laser sheath over a lead was difficult even after 5–6 s of laser ablation, crossover to the Evolution system was performed first rather than changing the target from one lead to another one. Additionally, in cases where the laser sheath could not be advanced, the same approach was used rather than switching to a larger‐bore laser sheath.

**FIGURE 1:**
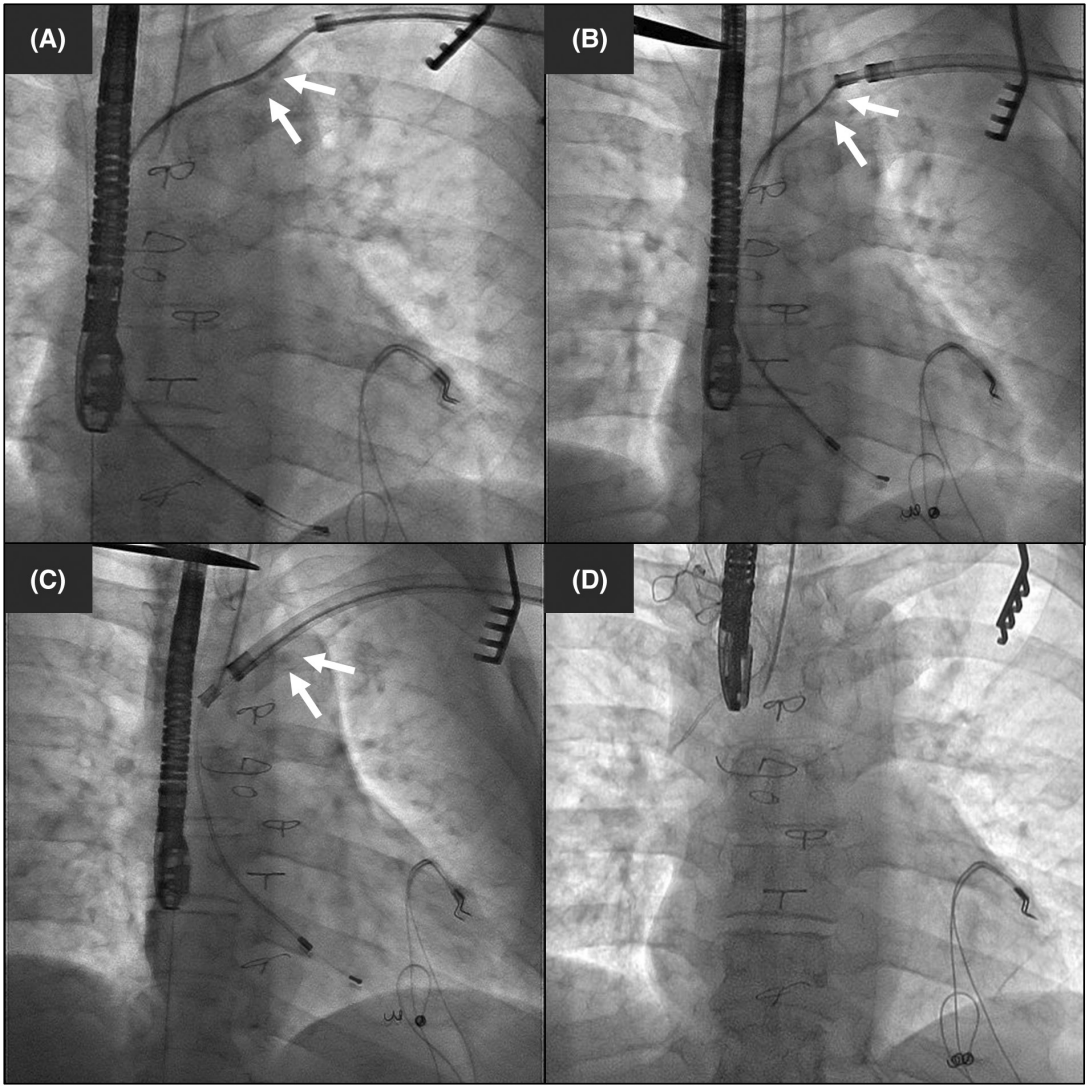
A representative case wherein crossover from a GlideLight laser sheath to an Evolution RL sheath was needed. (A) Lead extraction was started with a 14‐Fr GlideLight laser sheath. However, the sheath could not be advanced over the lead. Note that a fluoroscopically evident calcification lesion along the lead was present ahead of the laser sheath (white arrows). (B) The laser sheath was switched to an 11‐Fr Evolution RL sheath, and the sheath started to move forward little by little. (C) The Evolution RL sheath was advanced beyond the calcification lesion. (D) The lead was completely extracted.

The decision to switch back from the Evolution system to a laser sheath after dissecting the adhesion was made by the operators, and this switch was made because a laser sheath has a more flexible shaft. The more flexible shaft maintained the coaxial to the targeted lead and facilitated advancement in cases when the vascular structures were tortuous. Moreover, a tandem femoral–superior approach^10^ was attempted depending on the rail strength of the lead during the procedure. In cases wherein a lead could provide sufficient rail strength for the advancement of the laser sheath but a laser sheath could not be advanced over the lead despite laser ablation, crossover from a laser sheath to the Evolution system was usually performed before the tandem femoral–superior approach. However, in cases wherein a lead could not create a strong rail for advancing the laser sheath, the tandem femoral–superior approach was introduced before crossover to the Evolution system. In the tandem femoral–superior approach, leads that did not have a free end were pulled caudally using a Needle's Eye Snare (Cook Medical), and leads that had a free end were pulled caudally using an EnSnare kit (ev3.). Additionally, the tandem femoral–superior approach included the wire‐loop technique using a pigtail catheter or an Agilis NxT steerable introducer (Abbott).^11^ The internal transjugular approach was not included in this study because it was not practiced routinely at our institution. Additionally, TightRail rotating dilator sheaths (Philips) were not available during the study period and, therefore, were not used in this study. A representative case showing crossover to an Evolution RL sheath is shown in Figure 1 and Videos [Link], [Link].

### 2.4 Definitions of outcomes and complications

Procedural outcomes were defined according to the 2009 Heart Rhythm Society consensus statement.^7^ Procedure time was defined as the interval from the open incision to the wound closure. Major intraprocedural complications included procedure‐related deaths, hemothorax, pericardiocentesis, and the requirement for urgent surgery during the TLE procedure.

### 2.5 Statistical analyses

Continuous variables are presented as medians with interquartile ranges (IQRs) and categorical variables are presented as frequencies (percentages). Continuous variables were compared using Wilcoxon's rank‐sum test, and categorical variables were compared using Fisher's two‐tailed exact test. Associated factors of crossover were investigated using a univariate logistic regression analysis followed by a multivariate logistic regression model using all significant variables (<0.05) from the univariate analysis. Data from the univariate and multivariate analyses are presented as odds ratios (ORs) with 95% confidence intervals (CIs). ORs for continuous variables represent the relative increased risk of endpoint per unit increase. A two‐sided *p*‐value of <0.05 was considered significant. All statistical analyses were performed using the JMP software (version 14; SAS Institute Inc.).

## 3 RESULTS

**FIGURE 2:**
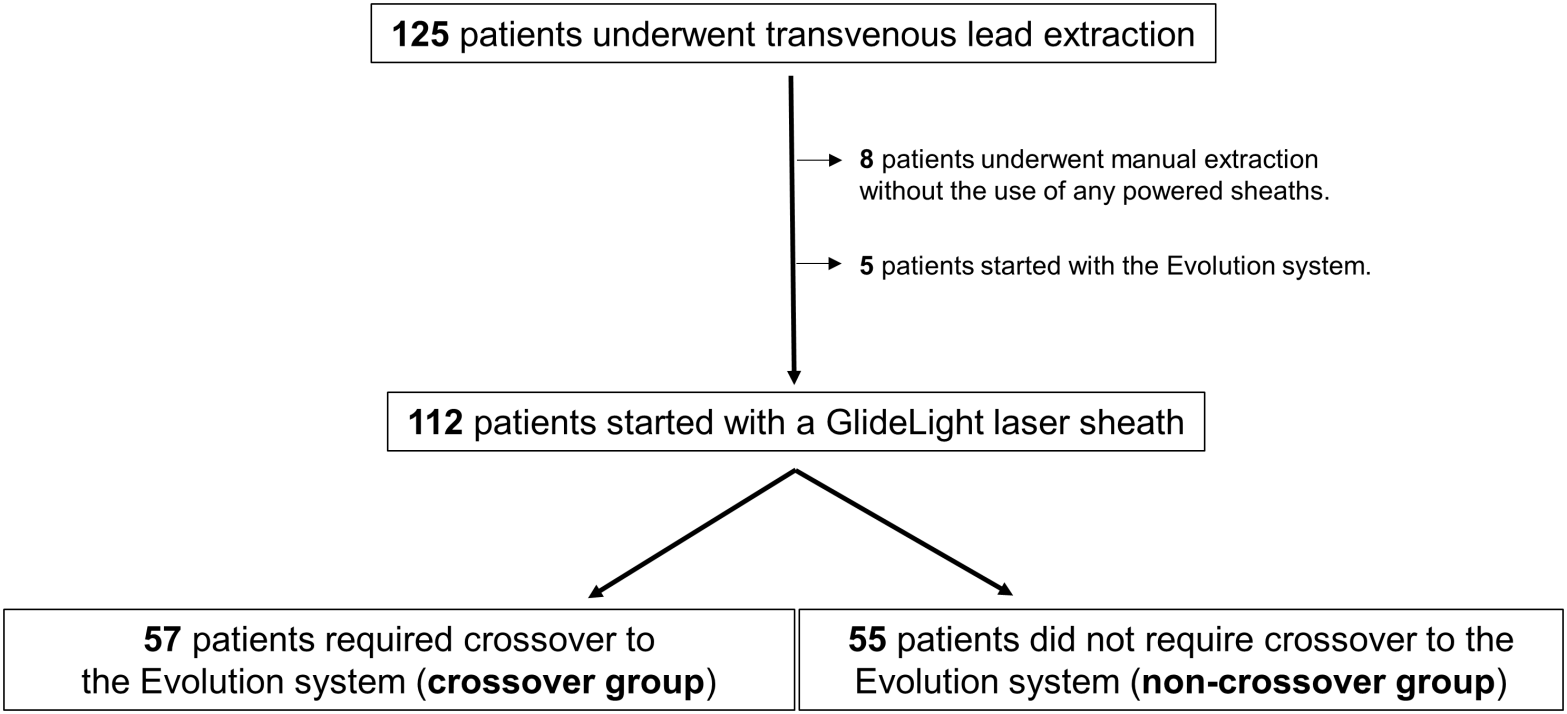
Study flowchart. Of the 125 consecutive patients undergoing transvenous lead extraction, a total of 112 patients were included in the analysis.

We screened 125 consecutive patients undergoing TLE. Eight patients were excluded because they underwent manual extraction of leads without any use of powered sheaths, and five patients were excluded because the Evolution system was used as the primary approach during their procedure. Consequently, 112 patients undergoing TLE were included in the analysis (Figure 2). Overall, 57 patients (50.9%) required crossover to the Evolution system (crossover group), and 55 patients (49.1%) did not require crossover (non‐crossover group). Table 1 shows baseline patient characteristics. No significant differences in baseline patient characteristics were observed between the crossover and noncrossover groups except for the male gender. Lead extraction was performed due to infection in 29 (50.9%) and 34 (61.8%) patients in the crossover and noncrossover groups, respectively. Table 2 shows the extracted lead characteristics and procedural characteristics. Dwell time of the oldest extracted lead was significantly longer in the crossover group. In addition, significantly more leads were extracted per procedure, the rates of apparent calcification of the encapsulating lead on fluoroscopy and passive fixation atrial/ventricular lead were significantly higher, and a femoral approach was used significantly more often in the crossover group than in the noncrossover group. In contrast, the rates of the failure of locking stylet in advancing to the lead tip in ≥1 pacemaker or implantable cardioverter defibrillator leads were not significantly different between the crossover and noncrossover groups. Moreover, patients with two or more leads were compared to those with a single lead to investigate the impact of lead‐to‐lead adhesion (Table S1). The frequency of device crossover from a laser sheath to the Evolution system was numerically higher in patients with two or more leads than those with a single lead; however, there was no significant difference between the frequencies.

**TABLE 1:** Baseline patient characteristics.

Variables	Overall *n* = 112	Crossover group *n* = 57	Non‐crossover group *n* = 55	*p* ^a^
Age, median (IQR), years	70.5 (63.3–81.8)	70.0 (62.0–83.0)	71.0 (64.0–80.0)	.97
Sex (male), *n* (%)	86 (76.8)	49 (86.0)	37 (67.3)	.025^b^
Body mass index, median (IQR), kg/m^2^	22.8 (20.4–25.4)	22.5 (20.3–25.1)	22.8 (20.4–25.8)	.65
Diabetes mellitus, *n* (%)	36 (32.1)	21 (36.8)	15 (27.3)	.32
Coronary artery disease, *n* (%)	18 (16.1)	12 (21.1)	6 (10.9)	.20
Previous cardiac surgery, *n* (%)	14 (12.5)	7 (12.3)	7 (12.7)	1.00
Stroke, *n* (%)	3 (2.7)	2 (3.5)	1 (1.8)	1.00
NYHA III or greater, *n* (%)	5 (4.5)	2 (3.5)	3 (5.5)	.68
Indication for extraction				
Infection, *n* (%)	63 (56.3)	29 (50.9)	34 (61.8)	.26
Access vein occlusion, % (*n*)	25.9 (28/108)^c^	26.8 (15/56)	25.0 (13/52)	1.00
Left ventricular ejection fraction, median (IQR), %	61.0 (43.0–70.0)	61.0 (39.0–71.5)	61.0 (45.0–69.0)	.78
Laboratory data on presentation				
Hemoglobin, median (IQR), g/dL	13.0 (11.7–14.4)	13.2 (11.9–14.4)	12.8 (11.3–14.4)	.38
eGFR, median (IQR), mL/min/1.73 m^2^	54.0 (39.7–70.3)	56.0 (43.2–73.0)	53.0 (34.7–68.6)	.24

*Note*: Data are presented as medians (interquartile ranges) or *n* (%) unless otherwise indicated.

Abbreviations: eGFR, estimated glomerular filtration rate; IQR, interquartile range; NYHA, New York Heart Association.

^a^ *p*‐Values are based on Wilcoxon's rank‐sum test or Fisher's two‐tailed exact test, as appropriate, for comparisons between the groups.

^b^ Significant difference between patients who do and do not require crossover.

^c^ Of the 112 patients included in the study, 108 underwent preprocedural contrast venography of the access veins.

**TABLE 2:** Extracted lead characteristics and procedural characteristics (per patient).

Variables	Overall *n* = 112	Crossover group *n* = 57	Noncrossover group *n* = 55	*p* ^a^
Dwell time of all leads, median (IQR), years	8.6 (4.6–11.5)	9.8 (7.8–14.2)	5.6 (2.8–9.7)	<.001^b^
Dwell time of the oldest extracted lead, median (IQR), years	8.9 (5.5–12.7)	10.8 (8.5–17.8)	5.8 (3.6–9.7)	<.001^b^
No. of leads extracted per procedure, median (range: min–max)	2 (1–4)	2 (1–4)	2 (1–4)	.025^b^
Failure of the locking stylet to advance to the lead tip in ≥1 pacemaker or ICD lead, *n* (%)	32 (28.6)	21 (36.8)	11 (20.0)	.061
Apparent calcification encapsulating the lead on fluoroscopy, *n* (%)	12 (10.7)	10 (17.5)	2 (3.6)	.029^b^
Lead fixation of atrial or ventricular leads				
Active fixation atrial pacemaker lead, % (*n*)	37.2 (32/86)^c^	27.7 (13/47)	48.7 (19/39)	.072
Passive fixation atrial pacemaker lead, % (*n*)	64.0 (55/86)^c^	74.5 (35/47)	51.3 (20/39)	.043^b^
Active fixation ventricular pacemaker lead, *n* (%)	42 (37.5)	18 (31.6)	24 (43.6)	.24
Passive fixation ventricular pacemaker lead, *n* (%)	37 (33.0)	25 (34.9)	12 (21.8)	.016^b^
Active fixation ICD ventricular lead, *n* (%)	33 (29.5)	16 (28.1)	17 (30.9)	.84
Passive fixation ICD ventricular lead, *n* (%)	11 (9.8)	7 (12.3)	4 (7.3)	.53
Coronary sinus lead, *n* (%)	15 (13.4)	7 (12.3)	8 (14.6)	.79
Crossover to an Evolution RL sheath or an Evolution Shortie RL sheath, *n* (%)	57 (50.9)	57 (100)	0 (0)	*N/A*
Femoral approach (supportive or bailout use), *n* (%)	36 (32.1)^d^	29 (50.9)	7 (12.3)	<.001^b^

*Note*: Data are presented as medians (interquartile ranges) or *n* (%) unless otherwise indicated.

Abbreviations: ICD, implantable cardioverter defibrillator; IQR, interquartile range; *N/A*, not applicable; TLE, transvenous lead extraction.

^a^ *p*‐Values are based on Wilcoxon's rank‐sum test or Fisher's two‐tailed exact test, as appropriate, for comparisons between the groups.

^b^ Significant difference between patients who do and do not require crossover.

^c^ Among the 112 patients included in the study, 86 patients had a history of implantation of atrial leads. Of these, active fixation atrial leads were used in 32 patients, and passive fixation atrial leads were used in 55 patients, respectively. One patient had both an active and a passive fixation atrial lead.

^d^ No patient underwent a femoral approach for powered sheath removal when a powered sheath could not be removed due to a stuck pacemaker lead. Among the 36 patients in the crossover group in whom a femoral approach was required, 27 underwent a femoral approach after the device crossover from a laser sheath to an Evolution RL sheath or an Evolution Shortie RL sheath.

The procedural outcomes and complications are presented in Table 3. The rates of achieving clinical success were not significantly different between the two groups (the crossover group, 98.3% vs. the noncrossover group, 100%; *p* = 1.00). However, the rate of achieving complete procedural success was significantly lower in the crossover group than in the noncrossover group (87.7% vs. 100%; *p* = .013). Procedure times were significantly longer in the crossover group than in the noncrossover group (median: 149.0 min; IQR: 119.0–215.0 min vs. median: 106.0 min; IQR: 89.0–126.0 min; *p* < .001).

**TABLE 3:** Procedural outcomes and complications.

Variables	Overall *n* = 112	Crossover group *n* = 57	Non‐crossover group *n* = 55	*p* ^a^
Clinical success, *n* (%)	111 (99.1)	56 (98.3)	55 (100.0)	1.00
Complete procedural success, *n* (%)	105 (93.8)	50 (87.7)	55 (100.0)	.013^b^
Procedure time, median (IQR), min	125.0 (100.0–153.0)	149.0 (119.0–215.0)	106.0 (89.0–126.0)	<.001^b^
Periprocedural transfusion requirements, *n* (%)	13 (11.6)	8 (14.0)	5 (9.1)	.56
Major intraprocedural complications				
Right atrial or right ventricular perforation, *n* (%)	2 (1.8)	2 (3.5)	0 (0)	.50
Superior vena cava tear, *n* (%)	0 (0)	0 (0)	0 (0)	*N/A*
Femoral hematoma requiring surgical drainage or transfusion, *n* (%)	3 (2.7)	2 (3.5)	1 (1.8)	1.00
Pocket hematoma requiring surgical drainage or transfusion, *n* (%)	8 (7.1)	5 (8.8)	3 (5.5)	.72

*Note*: Data are presented as medians (interquartile ranges) or *n* (%) unless otherwise indicated.

Abbreviations: IQR, interquartile range; *N/A*, not applicable.

^a^ *p*‐Values are based on Wilcoxon's rank‐sum test or Fisher's two‐tailed exact test, as appropriate, for comparisons between the groups.

^b^ Significant difference between the patients who do and do not require crossover.

Two major intraprocedural complications occurred in the crossover group; a right ventricular perforation occurred during lead reimplantation immediately after successful lead extraction and a right atrial perforation occurred just after lead extraction that required immediate open‐heart surgery. The latter was caused by the Needle Eye Snare, not the powered sheath introduced via a superior approach. The snare was used to capture the lead transfemorally as a bailout for lead elongation caused by the superior approach. The considerably elongated lead tangled with the snare. Several attempts to untangle the elongated lead caused the right atrial perforation.

**FIGURE 3:**
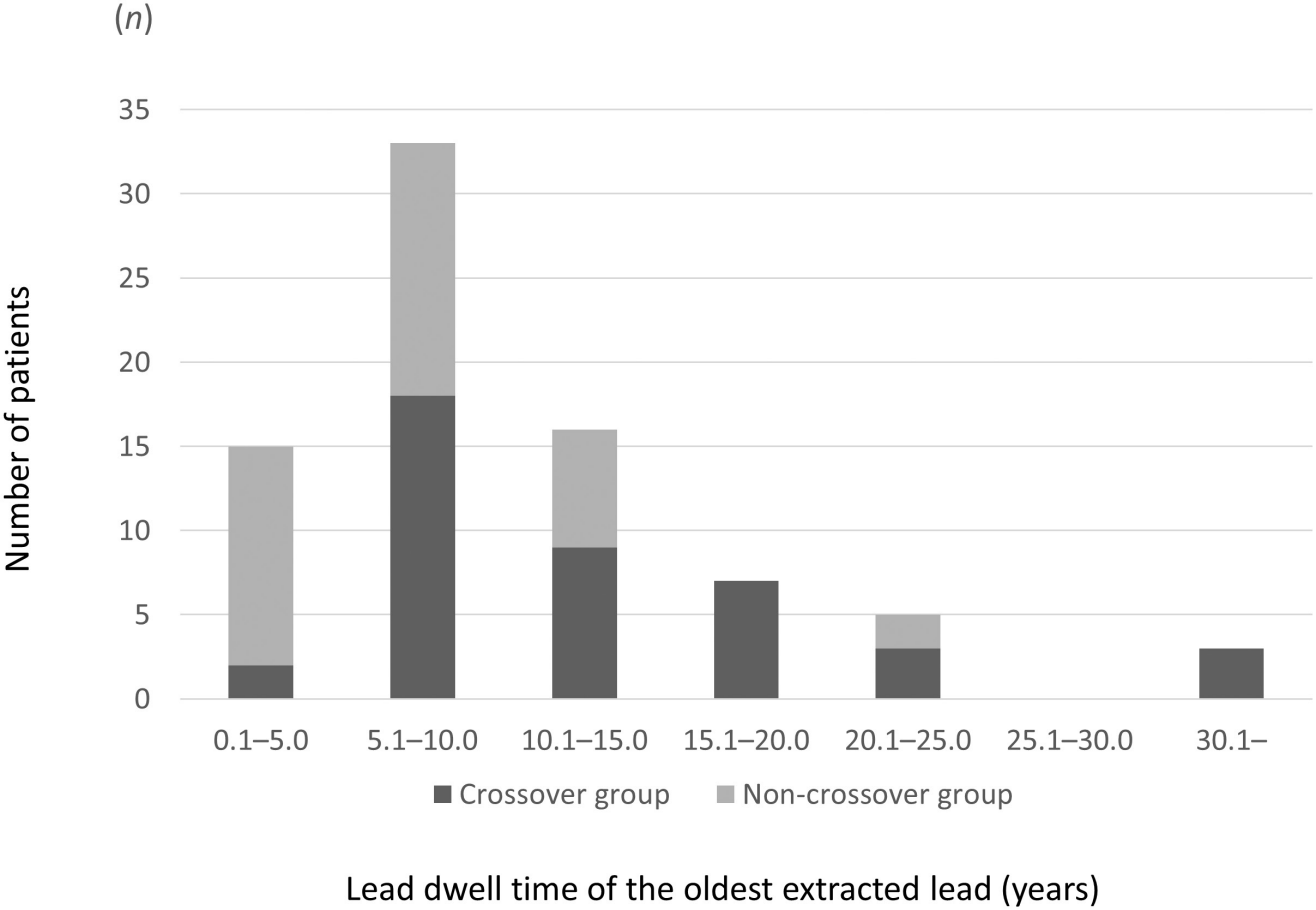
Distribution of lead dwell time for the oldest lead for patients requiring crossover and patients not requiring crossover from a laser sheath to the Evolution system. The rates of device crossover increased commensurately with the dwell time of the oldest extracted lead.

Multivariate analyses of clinical and lead‐related factors were performed to identify associated factors of crossover to Evolution system (Table 4). The dwell time of the oldest extracted lead (per year) (OR: 1.18, 95% CI: 1.02–1.36; *p* = .026), number of leads extracted per procedure (OR: 7.23, 95% CI: 1.74–29.99; *p* = .007), and use of a femoral approach (OR: 21.09, 95% CI: 2.33–190.67; *p* = .007) were significantly associated with crossover. The distribution of the lead dwell time of the oldest extracted lead and crossover to the Evolution system is displayed in Figure 3. The rates of device crossover increased in proportion to the dwell time of the oldest extracted lead. The receiver operating characteristic curve revealed that 7.7 years from implant was the cutoff period for the prediction of crossover to the Evolution system, with a sensitivity of 0.905 (90.5%) and a specificity of 0.649 (64.9%) (area under the curve: 0.80).

**TABLE 4:** Multivariate logistic regression analysis to determine associated factors of crossover to the Evolution system.

Variables	Adjusted OR (95% CI)	*p*
Dwell time of the oldest extracted lead, years	1.18 (1.02–1.36)	.026^a^
Number of leads extracted per procedure	7.23 (1.74–29.99)	.007^a^
Femoral approach (supportive or bailout use)	21.09 (2.33–190.67)	.007^a^
Sex (male)	2.13 (0.44–10.24)	.34
Apparent calcification encapsulating the lead on fluoroscopy	0.15 (0.01–2.53)	.19
Passive fixation atrial pacemaker lead	2.49 (0.68–9.12)	.17
Passive fixation ventricular pacemaker lead	1.90 (0.42–8.56)	.40

Abbreviations: CI, confidence interval; OR, odds ratio.

^a^ Significant association with crossover from a laser sheath to Evolution system (an Evolution RL sheath or an Evolution Shortie RL sheath).

## 4 DISCUSSION

To the best of our knowledge, this is the first study to demonstrate the safety and efficacy of timely crossover from a laser sheath to the Evolution system in cases of the unsuccessful initial use of a laser sheath. Additionally, both sheaths are leading contemporary powered sheaths; therefore, in this study, we investigated the safety and efficacy of the combined use of these sheaths. The results of this single‐center observational study reveal two important findings. First, longer dwell times for the oldest leads, more leads extracted per procedure, and the need for a femoral approach was associated with device crossover from a laser sheath to the Evolution system during TLE. Second, the rate of achieving clinical success was high in both (crossover and non‐crossover) groups when a crossover‐first strategy for switching to the Evolution system was chosen.

Longer dwell times of the oldest leads were associated with device crossover during TLE. Longer dwell times diminish the effects of the excimer laser system,^6^, ^12^ due to progressive time‐dependent tissue ingrowth or calcification along the lead.^13^, ^14^, ^15^ Progressive lead‐associated venous calcification is a barrier to lead extraction with a laser sheath. However, the calcification is difficult to detect on fluoroscopy. In fact, no significant differences in the incidence of fluoroscopically evident calcification on the lead were observed in our study, which may be due to the underestimation of calcification along the lead. Thus, even when no fluoroscopically evident calcification on the lead is detected, patients with old leads may require a crossover from a laser sheath to the Evolution system.

The significant association between the number of leads extracted per procedure and device crossover suggests that the incidence of lead‐to‐lead adhesions increases when more leads are implanted, requiring crossover during TLE. Lead‐to‐lead adhesions can hinder the advancement of a laser sheath. Moreover, lead‐to‐lead adhesions are a strong predictor of major complications; lead‐to‐lead adhesions cause a three‐fold increase in major complications during TLE.^16^ Thus, a switch to the Evolution system instead of a laser sheath could be a safe and effective choice for multiple lead extraction. Theoretically, when the crossover‐first strategy is adopted from the beginning of the TLE, the frequency of crossover from a laser sheath to the Evolution system should be significantly higher in patients with two or more leads than in those with a single lead. However, using a supportive femoral approach immediately after using a laser sheath may have eliminated the need for the Evolution system in some patients. Consequently, although the frequency of crossover from a laser sheath to the Evolution system was higher in patients with two or more leads than in those with a single lead in our study, this difference was not significant.

The need for a femoral approach during TLE was associated with device crossover. This result agrees with a previous study showing that a femoral snare was needed more frequently when using the Evolution system compared with the laser sheath,^17^ which may probably be due to a stiff shaft of the Evolution system. Pulling the lead downward using a femoral snare creates a strong rail for the advancement of the Evolution system. Additionally, a tandem femoral–superior approach, which incorporates snaring the targeted leads from a femoral approach combined with using the Evolution system advanced over the lead via a superior approach, effectively precludes superior vena cava injury; this approach reduces lateral forces when a powered sheath passes through the “hazard zone” of the innominate‐superior vena cava junction.^10^

The rate of clinical success was high and similar in both (crossover and noncrossover) groups. Thus, a strategy for crossover from a laser sheath to the Evolution system results in high rates of clinical success in complex lead extraction cases. The laser‐first approach is advantageous because the flexible shaft provides high coaxiality with the lead, which facilitates the introduction of the laser sheath into the implanted veins. In addition, the crossover‐first strategy reduces the risk of laser ablation injuries to implanted veins due to repeated activation of the laser sheath at the same spot.^18^ The ablative effects of the laser on calcified adhesions are minimal and consequently, the laser sheath could not be advanced over the calcified adhesions despite repeated ablation. Giving the laser more time and repeating ablation of the same spot in calcified adhesions increased the diameters of laser‐induced bubbles, which can lead to increased collateral damage and subsequent venous perforation. Therefore, not insisting on using a laser sheath for too long and the timely switch to the Evolution system may result in safe lead extraction.

### 4.1 Study limitations

This study has some limitations. First, this was a retrospective, observational, and nonrandomized study that included a limited number of patients at a single center. Thus, this study did not demonstrate the superiority of the Evolution system alone over the combination of a GlideLight laser sheath and the Evolution system. Second, the decision for device crossover is inherently operator‐dependent, although we tentatively decided the timing for crossover as a laser ablation of 5–6 s at the same spot. Third, device crossover may have underestimated the ability of laser sheaths in cases wherein multiple lead extractions were required. If the laser sheath did not advance over the initial lead, some lead extractors may have attempted to advance the laser sheath by switching back and forth between leads. However, in our study, the laser sheath was not initially moved back and forth between leads. In our opinion, despite the economic disadvantage imposed by the use of more medical devices, the greatest advantage of crossover from a laser sheath to the Evolution system before changing one target lead with another is the reduction in bleeding from the venous entry site in patients with two or more implanted leads. After the switch from the laser sheath, the venous entry site is almost completely plugged with the Evolution system, which controls bleeding from the venous entry site. In contrast, after the removal of the laser sheath to change the target from one lead to another, controlling bleeding from the venous entry site of the first target lead is difficult, because the venous entry site of the first target lead is not plugged with the laser sheath placed over the second target lead. Under this crossover‐first strategy, no significant differences in the incidence of periprocedural transfusion requirements and pocket hematoma requiring surgical drainage and transfusion were observed between patients with two or more leads and those with a single lead **(**Table S1). Fourth, the procedure time included the time required for TLE and that required for simultaneous implantation of new leads because these times were not separately recorded. However, among the 112 patients included in the present study, only 9 patients underwent TLE with simultaneous implantation of new leads. Thus, the procedure time was almost the same as that required for TLE. Finally, although polypropylene sheaths are useful for TLE,^19^ especially for calcified lesions in some cases, they were only used in one patient in the present study. A polypropylene sheath was used in this patient after crossover from a laser sheath to the Evolution system.

## 5 CONCLUSIONS

Dwell time of the oldest lead, the number of leads extracted per procedure, and the need for a femoral approach were associated with crossover from a GlideLight laser sheath to the Evolution system during lead extraction. High rates of clinical success were achieved in complex lead extraction cases when a crossover‐first strategy for switching to an Evolution system was employed. Therefore, switching to the Evolution system may facilitate safe and effective lead extraction when a laser sheath does not advance despite laser activation.

## FUNDING INFORMATION

No funding was received for conducting this study.

## CONFLICT OF INTEREST STATEMENT

The authors have no competing interests to declare that are relevant to the content of this article.

## ETHICS STATEMENT

All procedures in this study were performed in accordance with the ethical standards of the institutional research committee and the 1964 Helsinki Declaration and its later amendments or comparable ethical standards. The study was approved by the Institutional Research Committee of Sendai Kousei Hospital (approval number: 4–21; approval date: August 4, 2022).

## CONSENT STATEMENT

Written informed consent was obtained from all patients to publish this report.

## Supporting information


Table S1.

Video S1.

Video S2.

Video S3.

Video S4.

## Data Availability

The data used to support the findings of this study are available from the corresponding author upon request.
