# Supplementary material for: Associated factors and outcomes of crossover from a laser sheath to a bidirectional rotational mechanical sheath during transvenous lead extraction
Source: J Arrhythm. 2023 Sep 26;39(6):947–55. doi: 10.1002/joa3.12929 (PMC10692839; doi:10.1002/joa3.12929)
Supplement: Supplementary file 1 — Table S1. [file JOA3-39-947-s001.docx]

**Table S1.** Procedural characteristics, outcomes, and complications of the patients with two or more leads and those with a single lead

| **Variables** | **Overall**  ***n* = 112** | **Patients with two or more leads**  ***n* = 95** | **Patients with a single lead**  ***n* = 17** | ***P*^*^** |
| --- | --- | --- | --- | --- |
| Procedure time, median (IQR), min | 125 (100–153) | 133 (105–160) | 105 (77–131) | 0.009^†^ |
| Crossover to Evolution system, *n* (%) | 57 (50.9) | 52 (54.7) | 5 (29.4) | 0.068 |
| Clinical success, *n* (%) | 111 (99.1) | 94 (99.0) | 17 (100) | 1.00 |
| Complete procedural success, *n* (%) | 105 (93.8) | 88 (92.6) | 17 (100) | 0.59 |
| Periprocedural transfusion requirements, *n* (%) | 13 (11.6) | 12 (12.6) | 1 (5.9) | 0.69 |
| Pocket hematoma requiring surgical drainage or transfusion, *n* (%) | 8 (7.1) | 7 (7.4) | 1 (5.9) | 1.00 |

Data are presented as medians (interquartile ranges) or *n* (%) unless otherwise indicated. IQR, interquartile range

^*^*P*-values were determined using Wilcoxon’s rank-sum test or Fisher’s two-tailed exact test, as appropriate, for comparisons between the groups.

^†^Significant difference between the patients with two or more leads vs. those with a single lead.
